# Advanced Mathematical Platform for the Control and Manipulation of Magnetized Living Cells

**DOI:** 10.3390/bioengineering13050560

**Published:** 2026-05-15

**Authors:** Vitaly Goranov, Tatiana Shelyakova, Jaroslav Koštál, Alexander Makhaniok, Gianluca Giavaresi, Valentin Alek Dediu

**Affiliations:** 1 Institute for Nanostructured Materials, CNR-ISMN, 40129 Bologna, Italy; valentin.dediu@cnr.it; 2 BioDevice Systems s.r.o., Vršovice, 10100 Prague, Czech Republic; bowmathematical@gmail.com (J.K.); a.makhaniok@gmail.com (A.M.); 3 Surgical Science and Technologies, IRCCS Istituto Ortopedico Rizzoli, 40136 Bologna, Italy; gianluca.giavaresi@ior.it

**Keywords:** tissue engineering, magnetized cells, remote field control, COMSOL simulations, AI data analysis, machine learning

## Abstract

Magnetizing living cells with superparamagnetic iron oxide nanoparticles (SPIONs) enables their remote manipulation using external magnetic field. This lays the foundation for magnetically assembling tissue precursors within cell-friendly, proliferation-permissive environments and holds considerable promise for biomedical applications, particularly in the development of complex single- and multicellular tissue constructs for bone and organ reconstruction. However, progress in this field is limited by the lack of robust mathematical tools for accurate control of ensembles of magnetic nano- and micro-objects. In practical printing scenarios, collective behavior and unavoidable statistical heterogeneity—such as variations in SPION size and shape or deviations in cell magnetization—render traditional equation-based modeling inadequate. We developed a hybrid modeling framework integrating conventional physics-based simulations with artificial intelligence-driven image analysis. Dynamic parameters were extracted from video recordings of magnetized cells moving within model microfluidic devices exposed to well-defined magnetic fields and gradients. The AI-based analysis enabled quantitative characterization of ensemble behavior under heterogeneous conditions. The proposed framework successfully captured the collective dynamics of magnetized cell ensembles and enabled accurate control of their spatial organization under external magnetic actuation. The integration of simulation and data-driven analysis provided robust parameter identification despite statistical heterogeneity within the system. This integrated modeling approach provides a practical and effective tool for controlling the three-dimensional magnetic assembly of living cells, with strong potential for applications in tissue engineering.

## 1. Introduction

Regenerative medicine (RM) holds significant promise for advanced healthcare by offering personalized and specific approaches to tissue repair. Central to RM is the restoration and revitalization of damaged cells and tissues, presenting a potential revolution in managing various medical challenges. Despite advancements in the field and notable successes in specific cases, the full potential of regenerative medicine in clinical settings remains unrealized.

Progress in material science and engineering have enabled the realistic design and printing of complex tissue arrangements, aiming to achieve full-scale organ printing [1,2,3]. Nevertheless, bone treatments proceed so far through transplantation, which is the second most common transplant procedure after blood transfusion. Structurally and functionally adaptive artificial bone must incorporate stimuli-responsive materials compatible with 3D (bio)printing techniques and suitable biological and mechanical properties, a connected porous structure, and a shape matching the bone defect [4,5,6,7]. Traditional bioprinting approaches frequently fall short in reproducing the intricate cellular architectures and interactions essential for complex tissue formation. A key obstacle to that is the correct assembly of living cells into functional tissues. This challenge is particularly pronounced for tissues characterized by heterogeneous cell compositions and architectural gradients—such as the articular tissues, including meniscus, cartilage, etc. [8], where the cell supply issues and controlled migration of stem cells represent problems still waiting for efficient solutions. Accomplishing accurate cellular organization and ensuring optimal metabolic support in such tissues pose significant challenges, limiting the broader clinical implementation of 3D printing.

Magnetic technologies—typically relying on the manipulation of magnetic micro- and nanoparticles by spatially defined magnetic fields [9]—have already been established as a powerful platform for numerous biomedical applications [10]. These include targeted drug and gene delivery [11], magnetic hyperthermia for cancer treatment [12], scaffold-supported bone reconstruction [13], and the capture and concentration of biomarkers for early diagnostics [14], among others. In this context, the remote magnetic guidance of living cells for 3D assembly emerges as a versatile and promising technology for tissue prototyping. This approach offers a strongly competitive edge over conventional 3D bioprinting techniques in the ongoing effort to translate the remarkable scientific advances into clinical practice [15]. Magnetic 3D printing of functional tissues leverages magnetically (SPIONs) labeled cells, which can be precisely directed through remote magnetic control, offering several advantages with respect to other bioprinting techniques. Among the main advantages, the capability to generate hierarchical multi-type cellular architectures by fast (pseudo-spontaneous) assembling of living cells clearly stands out. Note that the remote guiding allows us to handle the cells in friendly environments and move them gently by acting exclusively on the magnetic component [16]. Magnetic nanoparticles exhibit intrinsic magnetic properties and, when incorporated into biomimetic scaffold structures, they can sustain osteoconduction, osteoinduction, and angiogenesis [17,18]. Noteworthily, thorough toxicological investigations showed low cytotoxicity values for concentrations of magnetic material not exceeding 100 pg/cell—fully functional for the activation of cells by realistic and safe remote magnetic fields [16,19].

Functionally correct on-demand assembling of cells of the same or of different types, to produce tissue precursors for the repair of various injuries requires suitable and accurate printing protocols. The motion of a single magnetic object in any defined magnetic gradient can be calculated by straightforward mathematics [20]. On the other hand, describing the motion of a large set of nanoparticles, typically characterized by only approximately known distributions of shapes, sizes and magnetization, represents a clear challenge for the direct equation-based approach. Recently a Machine Learning/Artificial Intelligence (ML/AI) method was successfully employed to predict the magnetization of SPIONs from the video tracking of the dynamics of magnetic particles dragged in micro-capillaries by magnetic gradients [21].

The aim of this study is to demonstrate that a combined mathematical approach, integrating standard COMSOL-based and AI analyses, provides an excellent descriptive framework for the remotely controlled motion of magnetized cells. Experiments are conducted in a versatile model system using standard microfluidic chips compatible with direct optical observation and fluorescence detection, while SPION-doped fibroblasts were selected as suitable and useful cell models.

## 2. Materials and Methods

### 2.1. Cell Magnetization

To magnetize fibroblast line 3T3-GFP enabled with green fluorescence cultivated under a standard culture condition, we used commercial Chemicell (Berlin, Germany) MAG/G-PAA SPIONs with an average total diameter of 20 nm and a magnetic core diameter of 10 nm. The magnetizing procedure was based on previously established protocols [16,22] and employed concentrations from 1 × 10^3^ to 5 × 10^6^ SPIONs/cell. Although other concentrations were also tested, this range constituted the main basis for analyzing the effects of magnetization on the cellular fluxes in magnetic field.

### 2.2. Experimental System

**Figure 1:**
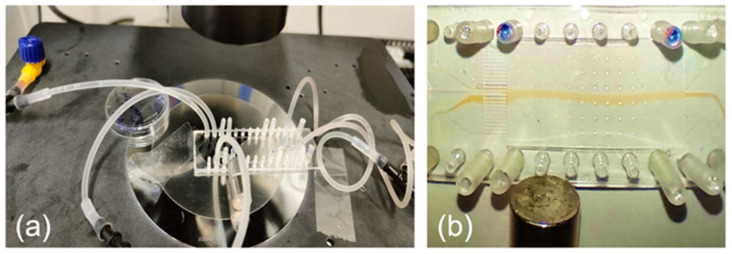
Experimental set-up. (**a**) The magnetic control and manipulation of all magnetic species is performed by a cylindrical NdFeB permanent magnet (10 × 10 mm^2^) positioned at 12 mm from the central axis of the chamber and at 25 mm downstream from the inlet. The magnet can be set in both upward and downward positions. (**b**) The formation of the laminar flow of the magnetized object’s flow and its deviation (downwards) by the magnetic field [23].

To analyze the flux of magnetized cells in magnetic fields, we used the ChipShop (Jena, Germany) 25 mm × 75 mm microfluidic chip coated with Zeonor (Zeon, Japan) polymer (Figure 1a). The chip has five inlets and an array of 25 narrow channels that subsequently merge into five wider outlet channels, optimizing fluid drainage and maintaining laminar flow regime. The cell suspension and buffer were introduced into the microfluidic network using a peristaltic pump to reduce the pulsation and ensure steady-state flows. This provides volumetric flow velocities within a range of a few tens up to several hundred micrometers per second in the central part of the chip and generates stable laminar flow conditions for the magnetized cells. Figure 1b shows the test flow of the SPION-based fluid, similarly to the previously published data [23].

To ensure reproducibility, experiments were conducted across four independent biological replicates (separate cell culture passages). For each replicate, eight technical independent microfluidic runs were performed under identical hydrodynamic and magnetic conditions. Magnetic deflection patterns were quantitatively assessed by measuring the lateral drift distance and deflection angle and extracting minimal intervals reflecting reliable flow-deviation from recorded video. The U-Net segmentation model maintained an IoU of 0.86 ± 0.05 across independent runs, confirming robust feature extraction despite minor optical or flow fluctuations. Elastic data augmentation further minimized sensitivity to run-to-run variability.

The dynamics of magnetized cells were monitored under a standard fluorescent Olympus IX71 microscope (Tokyo, Japan) equipped with a digital camera for video recording. A 10×–15× magnification was used to visualize the 100 × 100 pixel field in the chamber. Images and video sequences are recorded for data analysis with standard imaging software. Post-acquisition adjustments to brightness and contrast were also made to enhance the quality of visualization and enable a maximally precise assessment of the flow dynamics under the influence of the magnetic field. The recorded dynamics were analyzed using COMSOL 3.5a version and AI-based methods.

This setup has been already successfully validated on the flow of SPIONs, realized in similar conditions [23].

### 2.3. COMSOL Simulation Approach

Standard computer simulations were employed to model the transport behavior of magnetized cells. These simulations were carried out using COMSOL Multiphysics, specifically employing the Chemical Engineering Module with Mass Transport (Convection and Diffusion) and Transient Analysis.

The simulation environment consisted of a microfluidic chamber with dimensions: height = 175 µm, width = 14 mm, and length = 32 mm. A cylindrical permanent magnet (PM) with a diameter and height of 10 mm, and a residual magnetization of 1.3 T, was positioned horizontally and perpendicular to the chamber (Figure 1). The magnet was placed 5 mm (dist_y) from the nearest edge of the chamber (or 12 mm from the flow axis) and 25 mm (dist_x) from the chamber inlet. Magnetized cells, each with a diameter of 10 µm, were introduced into the chamber filled with a physiological solution having a dynamic viscosity of 1.1 mPa·s (1.1 × 10^−3^ N·s/m^2^).

The mathematical description in COMSOL is based on the balance of magnetic and fluid-dragging forces for magnetic particles:

$${\vec{F}}_{mag}+{\vec{F}}_{drag}=0,$$

where the magnetic force is given by:

$${\vec{F}}_{mag}=\mu_{0}M_{p}\nabla |\vec{H}|,$$

the dragging force is given by:

$${\vec{F}}_{drag}=-6\pi \eta r_{p}({\vec{v}}_{p}-{\vec{v}}_{f}),$$

and the particle velocity is:

$${\vec{v}}_{p}={\vec{v}}_{f}+\frac{{\vec{F}}_{mag}}{6\pi \eta r_{p}},$$

Here ${\vec{v}}_{f}$ is the fluid velocity, *η*—fluid dynamic viscosity, $M_{p}$—particle magnetization, and H is the applied field. The buoyancy force is ignored throughout the paper as non-relevant for the horizontal projection of the motion, detected in our configuration.

### 2.4. AI—Methodology

To move on complementary to COMSOL algorithms, we shall consider that the real magnetized cells contain nanoparticles with dispersions in size, shape, and aggregation state, which directly influence their net magnetic moment [24]. When SPIONs are internalized by cells, clustering within endosomes functionally behaves as a larger “magnetic inclusion” rather than isolated nanoparticles [25]. Reported intracellular iron concentration typically spans in the 1–5 pg Fe/cell interval, depending on SPION type, coating, and incubation protocol [26]. Thus, commonly employed mathematical models for magnetic nanoparticle dynamics can result in significant deviations from instrumentally validated parameters [27]. The complex behavior of magnetized living cells—influenced by factors like cytoskeletal interactions, membrane fluidity, and variable SPION internalization—is poorly described by standard magnetostatic and hydrodynamic equations alone [28].

Machine learning (ML) and artificial intelligence (AI) techniques were employed to evaluate the magnetization of cells by analyzing their response to a remote magnetic field. This approach characterizes the displacement patterns—specifically within regions affected by magnetic forces—formed by magnetically controlled microflows. Video recordings, captured via standard fluorescence microscopy, served as input data for the neural network model.

Using the microfluidic chip described above, we estimate the drift (deflection) of the magnetized microflow under the influence of the external magnetic field. The recorded video of the temporal dynamics of the deflection (drift) of the cells’ microflow under the influence of magnetic field served as a data set for ML processing.

To improve reproducibility of the AI-assisted analysis, the complete image-analysis workflow was defined as a sequential pipeline: video acquisition, frame extraction, region-of-interest selection, annotation, model training, segmentation validation, extraction of magnetic-flow descriptors, and conversion of these descriptors into an estimated relative SPION loading per cell. Raw fluorescence videos were first converted into individual image frames using Python/OpenCV. For each experimental condition, the same predefined region of interest was used, corresponding to the zone in which magnetic deflection was reproducibly visible and where the signal-to-background ratio was highest. This choice is consistent with our previous AI-based microfluidic workflow for magnetic micro- and nano-objects, where the video-fixation field was deliberately selected in the region showing the most pronounced magnetic displacement.

Videos were recorded at 50 fps under fluorescence microscopy as described above. From eight independent experimental series, approximately 1700 frames were extracted at regular intervals to capture the full temporal evolution of the magnetized cell flow. Each frame was manually annotated using LabelMe (Cambridge, MA, USA); the cell-flow region, corresponding to the green fluorescent plume, was outlined as a single foreground class, while the remaining image area was treated as background. Annotations were converted to the COCO format and checked for consistency before model training. LabelMe is a graphical annotation tool commonly used for creating image datasets, and COCO is a widely used object-detection and segmentation format that supports segmentation masks, making this annotation route suitable for reproducible semantic segmentation workflows [29]. The annotated images were divided into training and validation subsets, while a separate evaluation set of 847 frames was used for the final performance assessment reported in Section 3.3.

To perform training, several convolutional neural network libraries could be employed, including ResAt-UNet, MLP, SVM, FCN8, Bilateral Segmentation Network (BisNet), DRNet, and DFA-Net. Previously, a systematic comparison of these [21] showed that the standard U-Net [30,31] gave the highest mean Intersection over Union (IoU) and pixel accuracy for the present segmentation task. The adopted U-Net followed the standard encoder–decoder structure, with a contracting path composed of repeated 3 × 3 convolutions, ReLU activations, and 2 × 2 max-pooling operations, and an expanding path composed of up-convolutions, concatenation with the corresponding cropped feature maps, and repeated 3 × 3 convolutions followed by ReLU activation. This architecture was selected because it combines global contextual information with accurate localization of small or weakly contrasted image regions, which is essential for detecting magnetically induced displacement of fluorescent cell-flow patterns.

Training used stochastic gradient descent with high momentum of 0.9 and a pixel-wise softmax/cross-entropy loss function. To mitigate overfitting and compensate for the limited dataset, on-the-fly data augmentation was applied, including random rotations, horizontal and vertical flips, brightness and contrast adjustments, and elastic deformations. Elastic deformation was particularly important because the segmented object was a deformable microflow rather than a rigid particle. The checkpoint with the lowest validation loss was retained. The random seed and software settings were recorded to improve computational reproducibility. The learning rate was set to 1 × 10^−4^, the model was trained up to 100 epochs, and the checkpoint with the lowest validation loss was retained.

Segmentation quality on the held-out validation/test frames gave an IoU of 0.88 and recall, or sensitivity, of 0.86, consistent with the values reported in the main text. Comparable metrics were obtained on the independent evaluation set of 847 frames—supporting model robustness across separate experimental recordings where, to assess run-to-run variability, the training procedure was repeated with different random initializations.

Extraction of magnetic-response descriptors was conducted for each segmented frame. The binary mask of the cell-flow region was converted into quantitative descriptors: centroid position, lateral drift distance relative to the no-field or inlet trajectory, deflection angle, mask area, and temporal displacement rate. The principal descriptor used for magnetization estimation was the lateral magnetic deflection, calculated as the distance between the segmented-flow centroid under magnetic actuation and the reference centroid obtained from control or initial frames acquired before appreciable magnetic deviation. For each SPION concentration, this descriptor was averaged over valid frames and technical replicates, while standard deviation and coefficient of variation were used to quantify run-to-run reproducibility. Frames with poor fluorescence intensity, out-of-focus regions, flow interruption, or mask fragmentation were excluded according to predefined criteria: segmentation area below the minimum experimentally observed flow area, centroid discontinuity larger than one channel width between consecutive frames, or segmentation quality below the validation threshold.

This descriptor-based approach follows the physical logic established in the earlier AI study on magnetic micro- and nano-objects [21]: the observed displacement of the magnetic-object flux in a microfluidic chamber is determined by the balance between magnetic attraction and hydrodynamic drag. In that study, finite-element simulations were used to relate displacement to hydrodynamic radius or relative magnetization, and the AI analysis was then used to identify the displacement patterns in video frames. The same principle is applied here to magnetized cells: the AI model does not directly count intracellular SPIONs, but extracts the experimentally visible response of the cell ensemble to the magnetic field. The comparison is most meaningful above 10^5^ SPIONs/cell, where magnetic force exceeds hydrodynamic noise, local flow instability, optical uncertainty, fluorescence variability, and cell-to-cell loading heterogeneity. This threshold is consistent with the previous AI study, which found that AI-based evaluation of relative magnetization outperformed finite-element analysis for average magnetic-object loadings exceeding 10^5^ objects/cell, while slowly or weakly magnetized cells remained difficult to evaluate because of heterogeneous nanoparticle loading. Below this range, the magnetic displacement approaches the detection limit of the experimental system; therefore, AI estimation becomes unreliable or non-applicable, and COMSOL estimates become dominated by model assumptions rather than measurable deflection.

## 3. Results

### 3.1. Video Recordings

**Figure 2:**
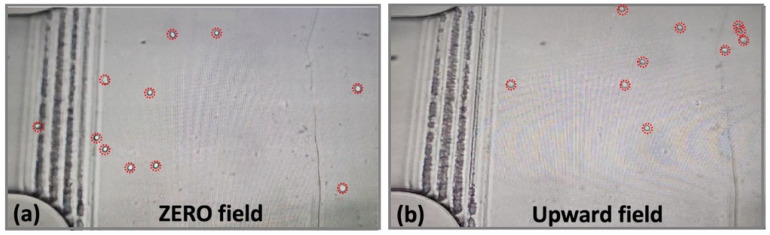
Raw data—microscopic observations of the low-concentration flow of magnetized cells—general top-down view. The cells are additionally evidenced by red circles, used as a guide for the eye.

**Figure 3:**
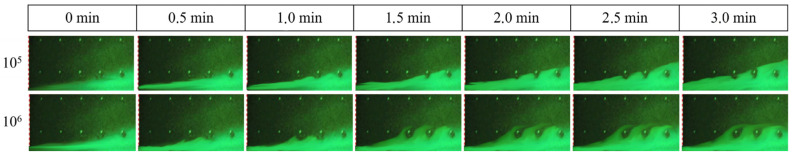
Raw data: microscopic observations of the high-concentration flow of magnetized cells in fixed 22.5 × 12 mm^2^ frames.

Figure 2 and Figure 3 show the laminar flow of the magnetized cells and its deviations caused by magnetic fields for low and high concentrations of cells respectively. Also, while Figure 2 displays the large-scale distribution of cells over tens of mm, Figure 3 is strongly zoomed on a few-mm optical frame.

The snapshots from the recorded videos in Figure 2 give a clear indication of a strong deviating effect of the magnetic field on highly magnetized cells (1 × 10^6^ SPIONs/cell). While in the absence of dragging magnetic field the cell flow is fairly uniform (Figure 2a), the presence of the permanent magnet, generating an upward force with respect to the image plane (Figure 2b), strongly deviates the magnetized cells towards the magnet, demonstrating evident remote cell-manipulating capabilities.

Figure 3 corresponds to snapshots from the starting time (0 min) to 3 min, taken at 0.5 min intervals. The top and bottom rows depict example figures for two chosen concentrations of 1 × 10^5^ and 1 × 10^6^ SPIONs/cell and for the sake of simplicity the figures are oriented so that the magnetic forces drag the cells upward, like in Figure 2. It can be seen that the green fluorescence pattern, corresponding to magnetized cells fluid, continuously diverges over time towards the magnet, and this deviation is more sizeable for higher concentrations.

Building on direct experimental observations, we now construct a proper mathematical treatment of the data, enabling us to provide a correct quantitative description of magnetic fluids and set the conditions for accurate 3D printing and assembling living cells.

### 3.2. COMSOL Simulations of Cell Motion

The motion of magnetized cells is simulated by considering cells as large cell-size microparticles borrowing a magnetic core, corresponding to the number of SPIONs inside the cell. To simplify the computer simulations, it is assumed that the whole magnetic material inside the cell is concentrated as a sphere with diameter d_mag, instead of considering specifically the MNP diameters, their number and their distribution.

**Figure 4:**
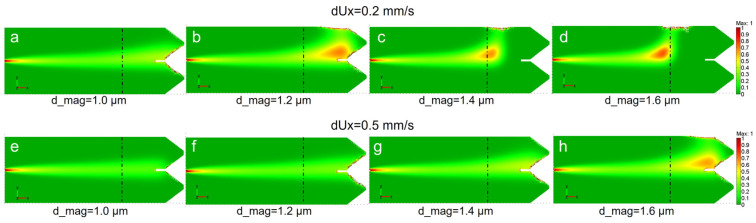
Distribution of the cell concentration for two fluid velocities dUx and different cell magnetic diameters d_mag: (**a**–**d**) dUx = 0.2 mm/s; (**e**–**h**) dUx = 0.5 mm/s; (**a**,**e**) d_mag = 1.0 µm; (**b**,**f**) d_mag = 1.2 µm; (**c**,**g**) d_mag = 1.4 µm; (**d**,**h**) d_mag = 1.6 µm. The PM axes indicated by dash–dot lines. Color scale from 0 to 1 mol/m^3^.

Figure 4 shows the results of the simulations for two fluid velocities, namely dUx = 0.2 mm/s (a–d) and 0.5 mm/s (e–h), and a range of magnetic diameters d_mag from 1.0 µm to 1.6 µm, corresponding to 1 × 10^6^–4 × 10^6^ internalized SPIONs. The distribution of the cell concentration is represented as a color scale from 0 (green) to 1 mol/m^3^ (red) and the position of PM axes is indicated by dash–dot lines. One can see that the magnetic fluid is sensitive to both magnetic core size and the fluid velocity—both parameters allow us to control and adjust the remote guiding and manipulation effect. For dUx = 0.2 mm/s and d_mag = 1.0 µm the flux only slightly turns toward the magnet. For bigger d_mag it strongly turns moving closer to PM axis. For higher velocity dUx = 0.5 mm/s this occurs only for the biggest d_mag = 1.6 µm, while for smaller diameters the flux only slightly modifies or does not change at all. Evidently, for low velocity fluid the vertical magnetic force strongly dominates over the horizontal dragging component, while the higher laminar velocity requires more magnetic material to induce a sizeable deviation. The flexibility of the remote magnetic guiding is confirmed by essentially similar distribution of fluids in Figure 4a,g and in Figure 4b,h, enabling efficient magnetic control through the competition of the two parameters. Note that the microfluidic environment represents an appropriate model research instrument, feasibly adaptable to 3D printing settings.

**Figure 5:**
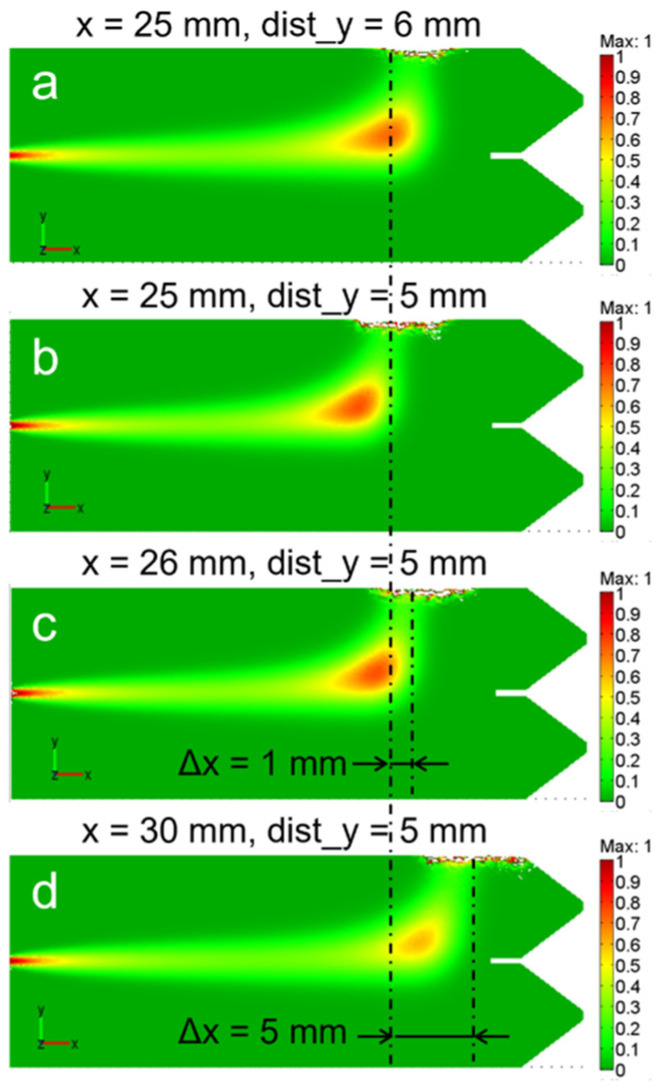
Distribution of the cell concentration for different magnet positions: (**a**) x = 25 mm, dist_y = 6 mm; (**b**) x = 25 mm, dist_y = 5 mm; (**c**) x = 26 mm, dist_y = 5 mm; (**d**) x = 30 mm, dist_y = 5 mm; where x—distance from the flux input to the PM axis; dist_y—distance from PM to the box edge for d_mag = 1.6 µm, dUx = 0.2 mm/s. The dash-dot lines indicate the PM axes. Color scale from 0 to 1 mol/m^3^.

To further investigate the guiding capability of the remote magnetic control, we show in Figure 5 the effects produced on the fluid motion and accumulation by the magnet repositioning, for fixed d_mag = 1.6 µm and dUx = 0.2 mm/s parameters. For the sake of clarity, the initial position is shown in Figure 5b, while Figure 5a corresponds to the magnet being moved away from the fluid by 1 mm (from 5 to 6 mm along y). Instead, Figure 5c,d represent the concentration distribution for PM shifted along the x axis by 1 mm (x = 26 mm) and 5 mm (x = 30 mm) respectively.

The motion of the magnet clearly results in a manifest flux rearrangement. The magnetized fluid follows the magnet motion with roughly 1:1 transmission coefficient, allowing for accurate remote control. Indeed, standard precision mechanical transmitters allow us to achieve a few of µm precision in magnet positioning, projecting this accuracy into the fluid control and confinement.

In summary, COMSOL simulations have proven effective in accurately modeling and predicting the behavior of magnetized nano- and microcarriers, recovering large-scale behavior observed in Figure 1b and Figure 2b for various magnetic fluids. COMSOL is perfectly applicable for monodisperse ensembles, but it can also handle polydisperse assemblies, provided the dispersion function is known.

However, real samples—such as magnetized living cells—tend to form far more complex aggregates, characterized by non-uniform distributions of magnetic material within cells of variable sizes. Accurately modeling such systems requires advanced, iterative approaches. Today, these challenges are increasingly addressed using artificial intelligence, particularly machine learning, which excels at developing and refining predictive models in complex, data-rich environments. The next paragraph demonstrates the great power and efficiency of AI approach for the treatment of highly dense cellular fluids.

### 3.3. AI Analysis of Detected Videos

As explained above, more than 1700 images from eight consecutive experimental sets were extracted and manually labeled for training. For training we used the stochastic gradient descent implementation with high momentum to ensure that many previously seen training samples determined the update in the current optimization step. The energy function was computed using a pixel-wise soft-max over the final feature map combined with the cross-entropy loss function, which effectively penalized deviations from the true label at each pixel position. To characterize the features of MC’s microflow deflection in the framed field area of the microfluidic chamber, the trained U-Net model was employed to analyze selected images from different experimental sets.

**Figure 6:**
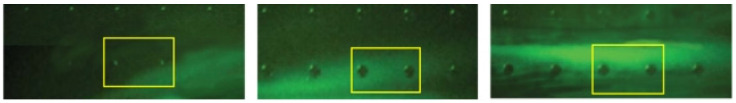
Fluorescence microscopic observations of the high-concentration flow of magnetized cells in fixed 22.5 × 12 mm^2^ frames—examples of quality-enhanced experimental recordings for 1 × 10^5^, 1 × 10^6^ and 5 × 10^6^ SPIONs/cell concentrations respectively (from left to right).

The rectangular regions of interest (ROI) outlined in Figure 6 were selected empirically from the microscopy recordings based on most reproducible magnetic deflections across experimental runs. These regions served as the primary input for the AI/ML pipeline, ensuring that training and validation frames contained the strongest signal-to-noise ratio for flow displacement and fluorescence contrast. By focusing on areas where the changes were mostly expressed, we maximized segmentation accuracy and temporal feature extraction, improving the reliability of the U-Net predictions while reducing error detections in peripheral, low-contrast regions.

Table 1 shows the estimates for cell magnetization by both COMSOL and AI approaches and their comparison with nominal experimental data. Here nominal concentration corresponds to the expected value, considering the amounts of the used materials (SPIONs and cells), the AI column stands for concentration calculated via ML methods, and the COMSOL column reflects the concentrations extracted from COMSOL calculations. Both methods rely on the same experimental data (magnetically induced flow deviation) but differ in implementation: direct data-driven inference versus model-based fitting.

In the AI-based approach, SPION loading was inferred from segmented video data by mapping experimentally measured magnetic-flow descriptors—primarily lateral deflection—onto the calibration relationship established between nominal loading and magnetic displacement. The “AI” column reports values averaged over valid frames and independent experimental runs, while the “COMSOL” column reflects simulation-based estimates under equivalent conditions. The approximation error was calculated as (estimated value − nominal value)/nominal value × 100%.

At high loadings (5 × 10^6^ and 1 × 10^6^ SPIONs/cell) corresponding to typical operating concentrations, the AI estimates show good agreement with nominal values: approximation error is about 8–10%; while COMSOL estimate errors are about 24% and 70% correspondingly. At cell magnetization of 5 × 10^5^ SPIONs/cell, the AI error increases to about 20%, remaining acceptable for semi-quantitative analysis. Starting from 1 × 10^5^ SPIONs/cell concentration and lower, the error rises sharply, reflecting the approach to the experimental detection limit, where magnetic deflection becomes comparable to noise and flow variability. Under these conditions, COMSOL estimates exhibit greater deviations, reflecting the heightened influence of model assumptions in low signal-to-noise regimes. Table 1 indicates that COMSOL modeling, while providing an efficient description for the large-scale fluid behavior (Figure 4 and Figure 5), is characterized by relatively low accuracy for the local sub-mm features. The failure of the method at very low concentrations respects the critical lowering of the magnetic force with respect to all competing effects.

The two methods, COMSOL and AI, are thus highly complementary to each other, providing a complete theoretical set for the system parametrization.

## 4. Discussion

The precise and gentle manipulation of living cells, particularly those functionalized with superparamagnetic iron oxide nanoparticles, represents a transformative approach for the 3D assembly of complex tissue constructs in regenerative medicine. Our study addresses a critical limitation in this field: the absence of robust mathematical tools for accurately controlling ensembles of magnetic nano- and micro-objects, especially given the inherent heterogeneity in SPION properties such as size, shape, and magnetization. Our hybrid framework, integrating experimental data, conventional COMSOL simulations, and AI-driven image analysis, provides a practical and effective solution to this challenge, establishing a robust modeling basis for the guided 3D magnetic assembly of living cells in bioprinting-relevant microfluidic environments [32].

COMSOL simulations appear to provide a comprehensive theoretical foundation for the description of the large-scale (chip size) transport dynamics of magnetized cells under remote field effects (see experimental data in Figure 1b and Figure 2). It shows that the microfluidic flow of the cells can be efficiently, and with good accuracy, controlled through the balance of magnetic and dragging forces. Indeed, at lower fluid velocities the magnetic deflection and concentration towards the permanent magnet are both very strong, while at higher fluid velocity the increased drag force partially neutralizes the magnetic influence, leading to a broader distribution. These achievements are crucial for optimizing bioprinting protocols, as they allow for the prediction of optimal flow rates and magnetic field gradients required to achieve desired cellular patterns and densities. Such predictive capabilities are essential for overcoming the challenges of recreating intricate cellular architectures and architectural gradients, which are often problematic for traditional bioprinting methods [33]. The ability to precisely control cell localization directly supports the goal of assembling hierarchical multi-type cellular structures. The level of control is crucial for the definition of spatial resolution and accuracy while constructing tissue precursors with specific architectural features, such as the cell density distribution in osteochondral tissues [34]. The near 1:1 correspondence between magnet displacement and fluid stream rearrangement revealed by COMSOL simulations (Figure 5) is promising and reveals the potential for cell-size precision accuracy in the positioning of cells in tissue aggregates. Indeed, considering the easily available tools for electro-mechanical control on the micro-scale and employing properly shaped permanent magnets [9], a 10–15 µm (cell size) spatial control looks totally feasible.

Note also that the magnetic force can be tuned by both the cell magnetization and by the magnetic field intensity-distribution, while the dragging force can also be tuned via two parameters—the fluid velocity and the viscosity. This endorses a high versatility for the control and modulation of the cellular fluids and the formation of bio-constructs.

The U-Net-based AI analysis represents a functional extension of the COMSOL modeling, advancing the mainly theoretical/predictive description of magnetic manipulation into a quantitative tool that operates in real time under realistic experimental conditions. U-Net-driven video analysis enables the extraction, at sub millimeter resolutions, of dynamic parameters of magnetized cellular microflows that are difficult to obtain with conventional methods, especially under weak signal, optical noise, cell overlap, and local density variations typical of microfluidic settings. Recent reviews on machine learning in microfluidics emphasize that combining physics-based modeling with data-driven image analysis significantly improves robustness when training data are limited and biological variability is high [31,35].

Our framework operationalizes this synergy: COMSOL provides the theoretical design space for magnetic actuation, and AI closes the loop by quantifying real-system deviations, enabling future closed-loop bioprinting control. We start from the experimental cell-flow pattern and end up with the cell magnetization amount responsible for the cellular motion in applied magnetic fields. The use of this approach in 3D printing will clearly require a reciprocal inversion of the modeling, where statistically defined magnetization and precisely defined magnetic gradients will bring the cell assemblies into desired cellular patterns or special positions.

Robust tracking of microflow deflection is not only a methodological advance but also supports translation to osteogenic bioprinting. The performance of bone bioconstructs strongly depends on the deposition-derived microarchitecture, including local distribution of osteoblasts/MSCs, controlled heterogeneity, spatial gradients, and preservation of filament geometry during layer-by-layer printing. Even small local deviations in flow trajectory or cohesion can yield hyper/hypocellular regions, interlayer discontinuities, and altered microenvironments (oxygen/nutrient diffusion and metabolite accumulation), ultimately affecting matrix deposition and mineralization. Here, U-Net’s ability to jointly capture global context and fine spatial details (via contraction/expansion paths and skip connections) is well suited to detect collective effects and micro heterogeneities that impact process reproducibility.

A further advantage is U-Net’s effectiveness with limited training data—common in biological studies. Data augmentation (including elastic deformations) improves robustness and invariance to morphological/kinematic variability, which is essential in bioprinting where bioink viscosity, temperature, batch variability, and flow/pressure fluctuations can alter flow dynamics and thus cell distribution within constructs.

Quantitatively, the AI approach accurately infers relative cellular magnetization for concentrations >10^5^ SPIONs/cell, outperforming standalone FEM simulations. Because magnetization directly links field/gradient design to patterning outcomes, improved estimation reduces systematic force prediction errors and increases reproducibility of bone-relevant strategies: cell enrichment in load bearing regions, density modulation near perfusable/vascularizable pores or channels, and precise interfaces in multi material or osteochondral constructs. Finally, reliable magnetization quantification helps balance manipulation efficacy with biological constraints, avoiding unnecessary nanoparticle loading; notably, the total iron oxide volume remains <1% of cell volume in the tested range, while still warranting dedicated assessment of oxidative stress, osteogenic phenotype, and long-term function.

Overall, the synergy between COMSOL and AI is central to the framework: COMSOL supplies a physically grounded, predictive model, while the AI extracts high resolution observables from the real system, effectively bridging the gap between theoretical predictions and actual behavior under complex experimental conditions. This integration supports a model-informed, data-driven approach to magneto-assisted bioprinting, where real time estimation of magnetization and flow dynamics can enable in process quality control and, ultimately, closed loop control (e.g., tuning process parameters or magnetic configuration to keep cell distribution within specifications). Key challenges remain, including achieving accurate quantification below 10^5^ SPIONs/cell—a desirable range to minimize biological impact—ensuring generalizability across different geometries and printing conditions, and establishing direct correlations between flow and magnetization metrics and osteogenic outcomes (e.g., ALP activity, matrix deposition, and mineralization) to validate the functional fabrication of bone constructs.

## 5. Conclusions

The advanced predictive capability presented in this paper is crucial for the progression of 3D magnetic bioprinting of living cells. By enabling precise control over magnetized cells within complex 3D microenvironments, our framework facilitates the assembly of intricate, hierarchical multi-type cellular architectures. This is an important step towards realizing the full potential of regenerative medicine, offering a pathway to address challenges such as controlled stem cell migration and the accurate organization of cells in complex tissues like articular cartilage or meniscus. The ability to remotely guide cells gently and precisely, acting exclusively on their magnetic component, represents a significant competitive advantage over conventional bioprinting techniques, as it minimizes mechanical stress and maintains a cell-friendly environment. Extrapolating our large-scale experimental results and their COMSOL treatment to the bioprinting context, the precision and versatility of the magnetic guiding look highly promising, and together with the two employed mathematical approaches constitutes a fairly complete technology.

## Figures and Tables

**Table 1:** COMSOL- and AI-based analysis of magnetized cellular fluids.

Nominal, SPIONs/Cell	AI, SPIONs/Cell	Approximation Error, %	COMSOL, SPIONs/Cell	Approximation Error, %
5 × 10^6^	4.6 × 10^6^	8	3.8 × 10^6^	24
1 × 10^6^	0.9 × 10^6^	10	1.7 × 10^6^	70
5 × 10^5^	6.0 × 10^5^	20	1.2 × 10^6^	140
1 × 10^5^	2.0 × 10^5^	100	7.0 × 10^5^	600
1 × 10^4^	-	-	1.0 × 10^5^	900
1 × 10^3^	Non applicable			
1 × 10^2^	Non applicable			

## Data Availability

Data is available from the corresponding author upon motivated request.
